# ELN risk stratification and outcomes in secondary and therapy-related AML patients consolidated with allogeneic stem cell transplantation

**DOI:** 10.1038/s41409-020-01129-1

**Published:** 2020-11-19

**Authors:** Madlen Jentzsch, Juliane Grimm, Marius Bill, Dominic Brauer, Donata Backhaus, Karoline Goldmann, Julia Schulz, Dietger Niederwieser, Uwe Platzbecker, Sebastian Schwind

**Affiliations:** Medical Clinic and Policlinic 1, Hematology, Cellular Therapy and Hemostaseology, Leipzig University Hospital, Leipzig, Germany

**Keywords:** Cancer immunotherapy, Acute myeloid leukaemia

## Abstract

Secondary or therapy-related acute myeloid leukemia (s/tAML) differs biologically from de novo disease. In general s/tAML patients have inferior outcomes after chemotherapy, compared to de novo cases and often receive allogeneic stem cell transplantation (HSCT) for consolidation. The European LeukemiaNet (ELN) risk stratification system is commonly applied in AML but the clinical significance is unknown in s/tAML. We analyzed 644 s/tAML or de novo AML patients receiving HSCT. s/tAML associated with older age and adverse risk, including higher ELN risk. Overall, s/tAML patients had similar cumulative incidence of relapse (CIR), but higher non-relapse mortality (NRM) and shorter overall survival (OS). In multivariate analyses, after adjustment for ELN risk and pre-HSCT measurable residual disease status, disease origin did not impact outcomes. Within the ELN favorable risk group, CIR was higher in s/tAML compared to de novo AML patients likely due to a different distribution of genetic aberrations, which did not translate into shorter OS. Within the ELN intermediate and adverse group outcomes were similar in de novo and s/tAML patients. Thus, not all s/tAML have a dismal prognosis and outcomes of s/tAML after allogeneic HSCT in remission are comparable to de novo patients when considering ELN risk.

## Introduction

Since acute myeloid leukemia (AML) is a biologically and clinically highly heterogeneous disease, a reliable risk stratification is very important to personalize treatment strategies. At diagnosis, the European LeukemiaNet (ELN) risk classification is a recommended risk stratification system, widely used, and has been shown to provide prognostic information in AML patients undergoing chemotherapy as well as allogeneic hematopoietic stem cell transplantation (HSCT) [1–3]. In addition, the evaluation of measurable residual disease (MRD) allows the adjustment of risk stratification during disease course [1, 4]. Over the last years, a growing incidence of patients with secondary (sAML) or treatment-related (tAML) AML has been observed [5, 6]. This comes as a result of the demographic changes with higher life expectancies as well as better cancer treatment options with an increasing number of patients surviving their primary neoplasm [6, 7]. The growing need for understanding s/tAML to improve risk stratification and subsequently patients’ outcomes is hampered by the low proportion of patients treated within clinical studies as compared to de novo cases [5, 8]. Regarding the associated prognosis, data on the rate of patients achieving a complete remission (CR) remain inconclusive with similar CR rates for de novo and tAML patients in a German analysis [7], but lower CR rates for s/tAML patients in Danish and Swedish registry data [8, 9] and another German study [2]. After consolidation chemotherapy, shorter disease free and overall survival (OS) have been observed for s/tAML compared to de novo cases [7–11]. The adverse outcomes of s/tAML were also suggested to be independent from the higher incidence of adverse risk cytogenetics, especially in younger AML patients [7–9, 12]. As a consequence of the low cure rates of not more than 20% after chemotherapy alone [8, 9], allogeneic HSCT often is the preferred consolidation option in s/tAML patients. Here, mostly registry-based data not including de novo AML individuals suggest allogeneic HSCT as a suitable and often curative treatment option for s/tAML patients [13–18]. However, data comparing outcomes of s/tAML and de novo AML patients undergoing allogeneic HSCT remain sparse. A recent registry-based analysis by the EBMT on reduced intensity (RIC) or myeloablative conditioning (MAC) HSCT showed higher relapse rates, higher non-relapse mortality (NRM), and shorter OS in s/tAML compared to de novo AML patients [19]. In contrast, a monocentric study in which the majority of patients were younger and received MAC suggested comparable outcomes for s/tAML patients [20]. However, s/tAML patients are often older than individuals with de novo AML [7–9], and may not be candidates for MAC- or even RIC-HSCT, also due to comorbidities and previous treatments. In addition, both studies did not report outcomes in the context of the current ELN risk classification, or the MRD status prior to HSCT, which both have been shown to impact patients’ outcomes [3, 21]. Here, we report outcomes of mostly older patients receiving allogeneic HSCT at our institution within the context of the most recent ELN risk classification.

## Subjects and methods

### Patients and treatment

We retrospectively analyzed 644 consecutive AML patients, who received an allogeneic HSCT at the University of Leipzig at a median age of 59.7 years (range 16.3–76.8 years). For all patients, associations of the disease origin with baseline clinical and genetic factors were assessed (“association set”). Of those, 534 patients were transplanted in CR or CR with incomplete peripheral recovery (CRi) and included in the outcome analysis (“outcome set”). Conditioning regimens in the 534 patients in the outcome set were either MAC (*n* = 142, 27%), RIC (*n* = 13, 2%) or NMA (*n* = 379, *n* = 71%). RIC conditioning was applied within the MC-FludT.14/L trial (EudraCT Number 2008-002356-18). Reasons for NMA-HSCT as opposed to MAC-HSCT were age over 50 years if receiving unrelated HSCT and over 55 years if receiving related HSCT, prior autologous HSCT (*n* = 7) or active infections (*n* = 8). All patients received G-CSF-stimulated peripheral blood stem cells as graft source. Stem cell donors were human leukocyte antigen (HLA) matched related (*n* = 121, 23%), HLA matched unrelated (*n* = 306, 57%) or had at least one HLA mismatch (*n* = 107, 20%). Prior to allogeneic HSCT, patients received age-dependent standard cytarabine-based chemotherapy protocols. As the reported patients received chemotherapy prior to the approval of a liposomal combination of cytarabine and daunorubicin (CPX-351) in Europe, none of the here analyzed s/tAML patients received the substance. Details on the applied therapies are given in the Supplementary Information. Further patients’ characteristics are shown in Table 1 and Supplementary Tables S1–S3. Median follow-up after HSCT was 3.7 years for patients alive. Written informed consent was obtained from all patients in accordance with the Declaration of Helsinki.

**Table 1:** Clinical and genetic characteristics for all patients according to disease origin (de novo vs secondary or treatment related), *n* = 644.

	All patients	De novo AML	Secondary or treatment-related AML	*P*
	*n* = 644	*n* = 416	*n* = 228	
Age at diagnosis, years				<0.001
Median	59.0	56.0	62.1	
Range	14.3–76.5	14.3–76.5	27.1–74.7	
Sex, *n* (%)				0.05
Male	334	204 (49)	130 (57)	
Female	310	212 (51)	98 (43)	
Hemoglobin, g/dL				0.69
Median	8.9	9	8.9	
Range	3.2–15.7	3.2–15.7	5.4–15	
Platelet count, ×10^9^/L				0.25
Median	63	65	59	
Range	1–950	2–950	1–547	
WBC, ×10^9^/L				0.01
Median	6.5	8.6	5.3	
Range	0.1–432	0.5–385	0.1–432	
Blood blasts, %				<0.001
Median	20	24	12	
Range	0–98	0–98	0–97	
BM blasts, %				<0.001
Median	50	60	35	
Range	0–100	0–100	0–95	
BM CD34+/CD38- burden, %				<0.001
Median	0.7	0.5	1.1	
Range	0–89	0–75	0–89	
Normal karyotype, *n* (%)		0.006		
Absent	329	192 (52)	137 (63)	
Present	259	180 (48)	79 (37)	
ELN2017 genetic risk group, *n* (%)				<0.001
Favorable	114	97 (33)	17 (12)	
Intermediate	129	84 (28)	45 (32)	
Adverse	195	115 (39)	80 (56)	
*NPM1*, *n* (%)				<0.001
Wild type	345	217 (71)	128 (86)	
Mutated	111	90 (29)	21 (14)	
*CEBPA*, *n* (%)			0.73	
Wild type	326	232 (88)	94 (87)	
Mutated	45	31 (12)	14 (14)	
*FLT3*-ITD, *n* (%)				<0.001
Absent	358	224 (72)	134 (90)	
Present	103	88 (28)	15 (10)	
*FLT3*-TKD, *n* (%)				0.003
Wild type	379	248 (87)	131 (96)	
Mutated	42	37 (13)	5 (4)	
*RUNX1*, *n* (%)				1
Wild type	95	61 (85)	34 (85)	
Mutated	17	11 (15)	6 (15)	
*ASXL1*, *n* (%)				0.41
Wild type	95	63 (88)	32 (80)	
Mutated	17	9 (13)	8 (20)	
*TP53*, *n* (%)				1
Wild type	99	64 (89)	35 (88)	
Mutated	13	8 (11)	5 (13)	

*ASXL1* additional sex combs-like 1 gene, *BM* bone marrow, *BAALC* brain and acute leukemia, cytogenetic gene, *CEBPA* CCAAT/enhancer-binding protein alpha gene, *ELN* European LeukemiaNet, *FLT3-ITD* internal tandem duplication of the FLT3 gene, *Hb* hemoglobin, *MN1* meningioma 1 gene, *NPM1* nucleophosmin 1 gene, *PB* peripheral blood, *RUNX1* Runt-related transcription factor 1 gene, *TP53* tumor protein 53 gene, *WBC* white blood count.

### Definitions of secondary or treatment-related AML

sAML was defined as AML developing after an antecedent myeloid neoplasm, i.e., myelodysplastic syndrome (MDS), myeloproliferative neoplasm (MPN), or MDS/MPN. tAML was defined as AML developing after exposure to chemotherapy or radiation applied for the treatment of lymphomas, solid tumors, or autoimmune diseases [22].

### Cytogenetics, molecular marker, flow cytometry, and MRD

Cytogenetic aberrations, surface antigen expression of common surface markers, mutations in the genes *NPM1* and *CEBPA* and the presence of *FLT3*-ITD were assessed in pretreatment bone marrow samples as previously described [23, 24]. For patients with material available, the mutation status of 54 genes included in the TruSight Myeloid Sequencing Panel (Illumina) was evaluated at diagnosis as previously described [24, 25]. Patients were grouped according to the ELN2017 risk classification [1] according to the diagnostic cytogenetic and molecular data available. Determination of the leukemic stem cell population at diagnosis and pre-HSCT MRD status was performed as previously described [24, 26–28].

### Statistical analyses

All statistical analyses were performed using the R statistical software platform (version 3.4.3) [29]. For further details see the Supplementary Information.

## Results

### Incidence of s/tAML

**Fig. 1:**
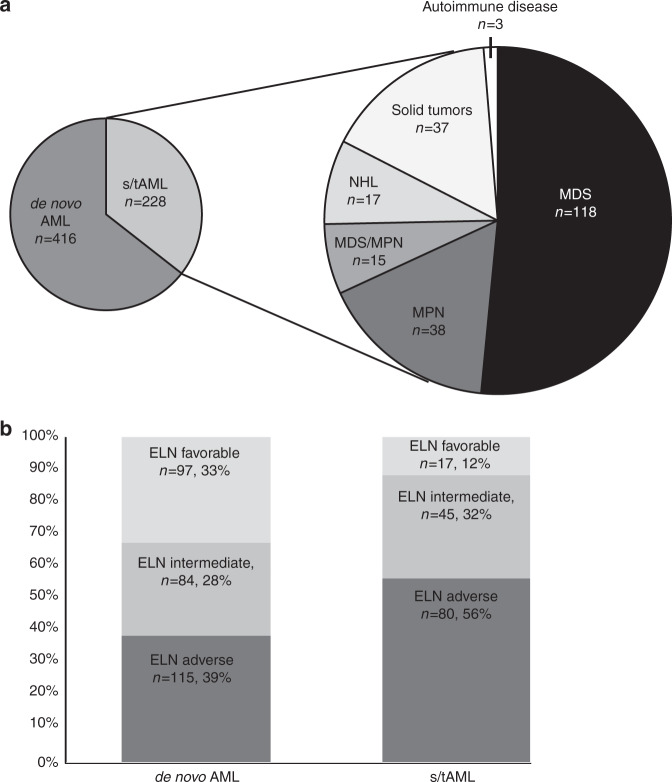
AML patients in the association set (*n* = 644). **a** Distribution of disease origin and **b** distribution of the ELN risk groups according to disease origin.

Overall, 416 patients (64%) had de novo AML while 171 patients (27%) had sAML (18% after prior MDS [MDS-sAML], 2% after prior MDS/MPN, and 6% after prior MPN [MPN-sAML]) and 57 patients (9%) had tAML (3% after lymphoma, 6% after a solid tumor and 0.5% of patients after autoimmune disorders, Fig. 1a). Detailed information on the type of prior malignancies is given in the Supplementary Information. Median time from cytotoxic treatment to AML diagnosis in tAML patients was 4.5 years (range 0.5–22.3 years).

### Characteristics of s/tAML patients

Compared to de novo AML patients, s/tAML patients were older (*P* < 0.001 and *P* = 0.006, for sAML or tAML, respectively) and had a lower white blood count (*P* = 0.03 and *P* = 0.05, respectively) at diagnosis (Table 1 and Supplementary Table S1). In addition, there were lower bone marrow (*P* < 0.001) and peripheral blood blast percentages (*P* < 0.001) at diagnosis and more male patients in the sAML patient cohort (*P* = 0.003). s/tAML patients also had a higher CD34+/CD38− cell burden (*P* < 0.001 and *P* = 0.05, respectively) and presented with a distinct immunophenotype (see Supplementary Information and Supplementary Table S2). s/tAML patients were more likely to have a del5/5q (*P* = 0.01 and *P* = 0.01, respectively) and a del7/7q (*P* = 0.05 and *P* < 0.001, respectively) but less likely to have a core binding factor (CBF) AML (*P* < 0.001 and *P* = 0.01, respectively), a normal karyotype (*P* = 0.05 and *P* = 0.02, respectively), or a *FLT3*-ITD (*P* < 0.001 and *P* = 0.05, respectively). In addition, patients with tAML more often had a complex (*P* = 0.006) [1] and a monosomal karyotype (*P* < 0.001) [30] while patients with sAML had a trend for more trisomy 8 (*P* = 0.06), were more likely to be *SRSF2* mutated (*P* = 0.03) or *JAK2* mutated (*P* < 0.001) but less likely to be *NPM1* (*P* < 0.001) or *FLT3*-TKD mutated (*P* = 0.001). Taken together, s/tAML patients were also by trend less likely to harbor *a RAS* pathway mutation (considering *NRAS*, *KRAS*, *HRAS*, and *PTPN11* mutations, *P* = 0.10). Regarding patients in the outcome set, sAML patients more often received a NMA conditioning (*P* < 0.001), were more likely to receive their allogeneic HSCT in first CR/CRi (*P* = 0.03), more likely to have a CRi compared to a CR (*P* = 0.005), while we observed no difference in pre-HSCT MRD status between de novo and s/tAML patients (*P* = 0.78, Supplementary Table S3). S/tAML patients were less likely to have a related donor (*P* = 0.009), by trend more likely to develop aGvHD (*P* = 0.06), while cGvHD was similar between s/tAML and de novo AML patients (*P* = 0.31). While tAML patients had a higher comorbidity index (HCT-CI) than de novo individuals (*P* < 0.001), the HCT-CI did not differ between de novo and sAML patients (*P* = 1). Importantly, s/tAML patients had a different distribution of the ELN risk groups compared to de novo disease and were more likely to harbor adverse ELN risk (*P* < 0.001 and *P* = 0.01, respectively, Fig. 1b),

### Outcome of s/tAML patients

**Fig. 2:**
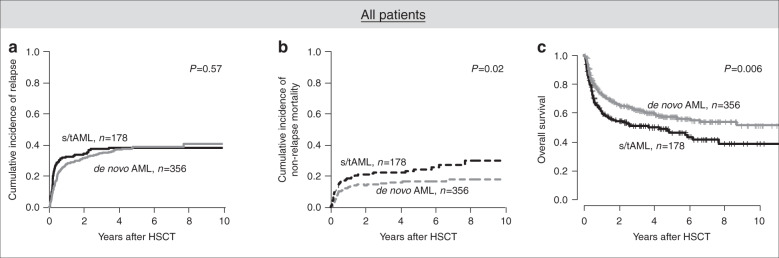
Outcome according to disease origin (de novo vs secondary or treatment-related AML) for patients in the outcome set (*n* = 534). **a** Cumulative incidence of relapse, **b** non-relapse mortality, and **c** overall survival in all patients.

**Fig. 3:**
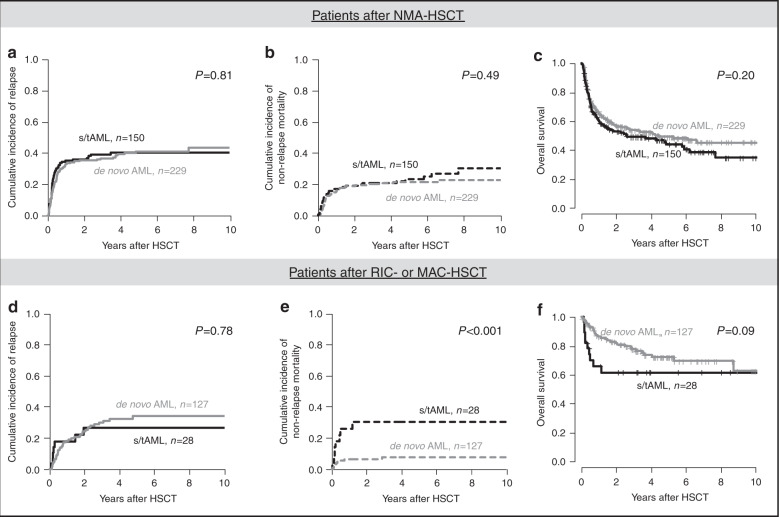
Outcome according to disease origin (de novo vs secondary or treatment-related AML) for patients in the outcome set (*n* = 534) given separately for both conditioning regimens. **a** Cumulative incidence of relapse, **b** non-relapse mortality, and **c** overall survival for patients receiving NMA-HSCT (*n* = 379) and **d** cumulative incidence of relapse, **e** non-relapse mortality, and **f** overall survival for patients receiving RIC- or MAC-HSCT (*n* = 155).

In the whole outcome set (Fig. 2), s/tAML patients had comparable CIR (*P* = 0.57) as de novo AML patients, but significantly higher NRM (*P* = 0.02) and shorter OS (*P* = 0.006). However, patients receiving NMA-HSCT had higher CIR (*P* = 0.02), higher NRM (*P* = 0.009), and shorter OS (*P* < 0.001) than patients receiving RIC- or MAC-HSCT (Supplementary Figs. S1 and S2) and a higher proportion of patients receiving NMA-HSCT had s/tAML (*P* = 0.03, Supplementary Table S3), resulting in a potential bias for outcome analyses. Thus, outcome was analyzed separately for NMA conditioned and RIC or MAC conditioned patients. Restricting the analysis to patients receiving NMA-HSCT (Fig. 3a–c), there was no different CIR (*P* = 0.81), NRM (*P* = 0.49), or OS (*P* = 0.20) between de novo and s/tAML patients. In contrast, in patients receiving RIC- or MAC-HSCT (Fig. 3e, f) with the caveat of limited patient numbers (*n* = 28) and a potential selection bias, s/tAML patients had a significantly higher NRM (*P* < 0.001), by trend shorter OS (*P* = 0.09) but similar CIR (*P* = 0.78). Similar results were observed when we restricted our analyses to patients transplanted in first CR/CRi (Supplementary Fig. S3).

In multivariate analyses for the whole patient cohort, s/tAML patients did not have distinct outcome compared to patients with de novo AML while ELN risk and pre-HSCT MRD status remained significant factors for CIR and OS (Table 2). Multivariate analyses for patients receiving NMA-HSCT or RIC- or MAC-HSCT separately is shown in Supplementary Table S4.

**Table 2:** Multivariate analyses for all patients.

	Cumulative incidence of relapse		Cumulative incidence of non-relapse mortality		Overall survival	
	HR^a^ (95% CI)	*P*	HR^a^ (95% CI)	*P*	OR^b^ (95% CI)	*P*
ELN2017 genetic risk (adverse vs intermediate vs favorable)	1.72 (1.22–2.42)	0.002	–	–	0.66 (0.49–0.88)	0.006
Age at HSCT	–	–	1.03 (1.02–1.05)	<0.001	–	–
Remission status at HSCT (CR vs CRi)	–	–	0.46 (0.29–0.73)	0.03	–	–
Pre-HSCT MRD status (positive vs negative)	3.22 (1.89–5.48)	<0.001	–	–	0.54 (0.34–0.87)	0.01

Variables considered in the models were those significant at *α* = 0.10 in univariable analyses.

For cumulative incidence of relapse endpoint, variables considered were: ELN2017 genetic risk group, age at HSCT, and pre-HSCT MRD status conditioning regimen (RIC/MAC vs NMA).

For non-relapse mortality endpoint, variables considered were: disease origin (de novo vs s/tAML), age at HSCT, remission status at HSCT (CR vs CRi), conditioning regimen (RIC/MAC vs NMA), and donor type (mismatched vs matched unrelated vs related).

For OS endpoint, variables considered were: disease origin (de novo vs s/tAML), ELN2017 genetic risk group, age at HSCT, pre-HSCT MRD status, conditioning regimen (RIC/MAC vs NMA), remission status at HSCT (CR vs CRi) and donor type (mismatched vs matched unrelated vs related).

*AML* acute myeloid leukemia, *CI* confidence interval, *CR* complete remission, *CRi* complete remission with incomplete peripheral recovery, *ELN* European LeukemiaNet, *HSCT* hematopoietic stem cell transplantation.

^a^HR, hazard ratio, <1 (>1) indicate lower (higher) risk of relapse for the first category listed for the dichotomous variables.

^b^OR, odds ratio, <1 (>1) indicate lower (higher) chance of survival for the first category listed for the dichotomous variables.

### Clinical and genetic characteristics of s/AML patients within the three ELN risk groups

**Fig. 4:**
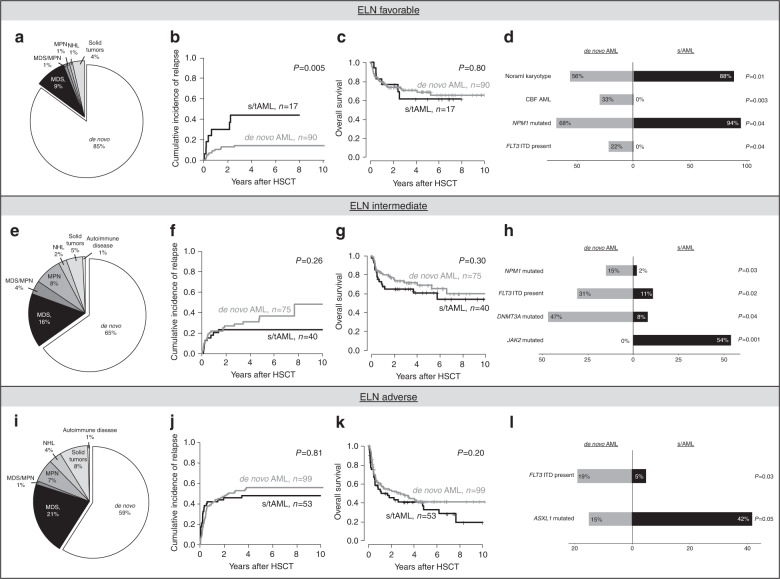
Outcome and disease characteristics according to disease origin (de novo vs secondary or treatment-related AML) within the separate ELN risk groups. **a** Distribution of primary disease, **b** cumulative incidence of relapse, **c** overall survival, and **d** genetic associations in patients with favorable ELN risk, **e** distribution of primary disease, **f** cumulative incidence of relapse, **g** overall survival, and **h** genetic associations in patients with intermediate ELN risk and **i** distribution of primary disease, **j** cumulative incidence of relapse, **k** overall survival, and **l** genetic associations in patients with adverse ELN risk.

Distribution of de novo or s/tAML as well as of primary neoplasm within s/tAML patients differed between the three ELN risk groups and is depicted in Fig. 4a, e, i.

Within patients with favorable risk according to ELN, s/tAML patients were older (*P* = 0.02) and had by trend a lower bone marrow blast percentage at diagnosis (*P* = 0.08) than de novo individuals. They were more likely to have a normal karyotype (*P* = 0.01) and to be *NPM1* mutated (*P* = 0.04), but less likely to harbor CBF AML (*P* = 0.003), or a *FLT3*-ITD (*P* = 0.04, Fig. 4d, Supplementary Table S5). Within patients with intermediate risk according to ELN, s/tAML patients were older (*P* = 0.02) and had lower bone marrow blast percentages at diagnosis (*P* = 0.003) than de novo individuals. They were less likely to be *NPM1* mutated (*P* = 0.03), to harbor a *FLT3*-ITD (P = 0.02) and to be *DNMT3A* mutated (*P* = 0.04) but more likely to be *JAK2* mutated (*P* = 0.001, Fig. 4h). Within patients with adverse risk according to ELN, s/tAML patients were older (*P* = 0.008), had lower platelet counts (*P* = 0.03), lower bone marrow blast percentages (*P* = 0.006) and a higher CD34+/CD38− cell burden at diagnosis= (*P* = 0.05) than de novo individuals. They were also less likely to harbor a *FLT3*-ITD (*P* = 0.03) and by trend *FLT3*-TKD (*P* = 0.09) and more likely to be *ASXL1* mutated (*P* = 0.05, Fig. 4l).

### Outcome of s/tAML patients within the three ELN risk groups

The ELN risk groups have been shown to allow a separation of patients in risk groups with distinct outcomes [2, 3, 31] and were distributed significantly different between de novo and s/tAML patients. Subsequently, we analyzed the prognostic impact of s/tAML compared to de novo AML within the ELN risk groups separately. Within the group of favorable ELN risk (*n* = 107, Fig. 4b, c), patients with s/tAML (*n* = 17) had significantly higher CIR (*P* = 0.005), but comparable OS (*P* = 0.80) as de novo AML patients (*n* = 90). Noteworthy is the low number of patients in the s/tAML group with ELN favorable risk, suggesting the results to be interpreted with caution. In contrast, neither within the group with intermediate (*n* = 115, Fig. 4f, g) nor adverse ELN risk (*n* = 152, Fig. 4j, k) distinct outcomes according to disease origin were observed. Finally, also in the high-risk group of patients with detectable pre-HSCT MRD, no distinct CIR (*P* = 0.63) and OS (*P* = 0.40, Supplementary Fig. S4) were observed.

## Discussion

The here observed associations of s/tAML compared to de novo AML patients are in line with previously published data [7–9, 12, 20]. Our study also shows that ELN adverse risk is more frequent and ELN favorable risk less frequent in s/tAML (Fig. 1b) compared to de novo AML patients.

After consolidation chemotherapy, adverse outcomes for s/tAML compared to de novo individuals have been shown, but this difference is reduced in older individuals (>60 years) or when high-risk genetic subgroups were regarded separately [7–9]. After allogeneic HSCT, there are only limited and conflicting data comparing de novo and s/tAML [19, 20] and no study focused on older individuals, representing the majority of s/tAML patients, and within the context of the most recent ELN risk classification. Regarding all patients, we observed a shorter OS for s/tAML patients which primarily was caused by higher NRM after allogeneic HSCT (Fig. 2b). Importantly, in multivariate analyses, after adjustment for ELN risk and pre-HSCT MRD status, disease origin did not impact CIR or OS. In separate analyses according to the applied conditioning regimens, no outcome difference between de novo and s/tAML was seen after NMA conditioning. Only within patients receiving RIC- or MAC-HSCT, having s/tAML remained a prognostic factor for higher NRM in both univariate and multivariate analyses. However, only 28 s/tAML patients received RIC- or MAC-HSCT in the here analyzed set. Two other studies compared de novo and s/tAML patients undergoing allogeneic HSCT [19, 20]. Overall outcomes in both studies match our analysis which is also true for the higher age and a more frequent use of lower intensity conditioning in the s/tAML cohorts. One other single centre analysis showed no distinct outcomes for patients transplanted in CR1/CRi1 [20]. In this study, lower patient numbers and no distinct cytogenetic risk between de novo and sAML patients may have contributed to the lacking outcome differences.

In contrast, the EBMT recently reported higher CIR and NRM and shorter OS for s/tAML patients, independently of conditioning intensity or cytogenetic risk [19]. Among the suggested reasons for these outcome differences was a lower ability to tolerate allogeneic HSCT-related toxicities in s/tAML patients. As we did not observe higher NRM or shorter OS after NMA-HSCT, but after RIC- or MAC-HSCT, our study contributes to this assumption of a predisposition to treatment-related complications after more intensive conditioning regimens in s/tAML patients. Another speculation of Schmaelter et al. was that a higher pre-HSCT MRD burden might have contributed to the worse outcomes [19]. We were able to assess the pre-HSCT MRD status (as previously described [26–28] based on *NPM1* mutation status and *BAALC* and *MN1* expression) in 244 patients which did not differ between de novo or s/tAML in our cohort, neither within the whole patient population (Supplementary Table S3) nor separately within the three ELN risk groups (Supplementary Table S5). As expected, MRD positivity correlated well with higher relapse probabilities which was seen irrespective of disease origin in both de novo and s/tAML patients (Supplementary Fig. S5) or conditioning regimen (Supplementary Fig. S6). In addition, outcome of pre-HSCT MRD positive patients was dismal and did not differ between de novo or s/tAML groups (Supplementary Fig. S4).

After consolidation chemotherapy, outcome differences between s/tAML and de novo AML patients have also been shown within different genetic risk groups [9, 32] but were reported to be larger in patients with favorable rather than adverse or intermediate genetic risk [12]. To our knowledge, we are the first to report on s/tAML patients receiving HSCT in the context of the most recent ELN risk classification, which relies to a larger extend on the molecular disease characterization [1]. Within the three ELN risk groups, between de novo and s/tAML patients, we observed a significantly different distribution of genetic characteristics, indicating distinct genetic drivers of the disease (Fig. 4d, h, i). In the ELN favorable group, s/tAML patients had less *FLT3*-ITD and CBF AML than de novo AML patients. Subsequently, and in contrast to the whole patient cohort, in ELN favorable risk the amount of patients with a normal karyotype or a *NPM1* mutation was higher in s/tAML than de novo AML patients. Within the ELN intermediate group, s/tAML patients were more likely to be *JAK2* mutated but less likely to be *NPM1*, *DNMT3A*, or *FLT3*-ITD mutated. In ELN adverse risk, s/tAML patients again had less *FLT3*-ITD, but were more often *ASXL1* mutated, which has been linked to sAML [33]. Regarding outcomes, only within the ELN favorable risk group we observed a higher CIR for s/tAML patients, likely driven by the lower incidence of CBF AML. CIR and OS remained similar between de novo and s/tAML patients within the ELN intermediate and adverse groups. Our data suggest that when the ELN risk groups are considered, no distinct survival can be shown between de novo or s/tAML patients receiving HSCT in remission and, thus, that allogeneic HSCT might contribute to better outcomes in this patient population.

Recently, a variety of new substances have been introduced into AML treatment. CPX-351 has been shown to improve outcomes for patients with s/tAML as compared to standard 7 + 3 chemotherapy [34]. Combination therapies of standard 7 + 3 with FLT3 inhibitors, as Midostaurin, in patients with *FLT3*-mutated AML [35] or gemtuzumab ozogamicin (GO) in CD33-positive favorable or intermediate risk AML [36] represent a new standard of care. Of note, none of the patients in our set received CPX-351 or GO and all patients treated within FLT3 inhibitor studies had de novo disease as prior chemotherapies were excluded according to study protocols. We also found lower CD33 expression levels in s/tAML compared to de novo AML patients (Supplementary Fig. S7), which might indicate reduced efficacy of GO in these patients and raises the question of applying higher dosages in selected patients. How these new substances will fit into the treatment of s/tAML patients remains to be elucidated, but treatment combinations of CPX-351 with GO (ClinicalTrials.gov Identifier: NCT03904251) and/or FLT3 inhibitors (NCT04128748) in eligible patients will likely further improve outcomes in s/tAML patients.

Relevant limitations of our study are the retrospective nature and restricted patient numbers within some subgroup analyses, including e.g., patients receiving RIC- or MAC-HSCT. In addition, only a restricted number of patients had the pre-HSCT MRD status available as well as could be comprehensively molecularly characterized for their *ASXL1*, *RUNX1*, and *TP53* mutation status—relevant for the genetic risk classification according to ELN2017 at diagnosis, leading to restricted patient numbers in some subgroups. Especially the s/tAML ELN2017 favorable subgroup included only 17 patients in the outcome analysis.

In conclusion, consistent with previous studies [19, 20], our data show that allogeneic HSCT is a feasible and often curative consolidation option for s/tAML patients. While s/tAML patients were older and more likely to harbor adverse ELN risk, outcomes between de novo and s/tAML patients did not differ when these covariables were considered. Thus, not all s/tAML patients have a dismal prognosis when undergoing allogeneic HSCT. Pre-HSCT MRD positivity remained an important prognostic factor in both de novo and s/tAML patients and showed no distinct incidence between both patient populations. These data highlight the importance of the ELN2017 classification and pre-HSCT MRD status for risk stratification also in s/tAML.

## Supplementary information

Supplemental Material
