# Supplementary material for: ELN risk stratification and outcomes in secondary and therapy-related AML patients consolidated with allogeneic stem cell transplantation
Source: Bone Marrow Transplant. 2020 Nov 19;56(4):936–45. doi: 10.1038/s41409-020-01129-1 (PMC8035074; doi:10.1038/s41409-020-01129-1)
Supplement: Supplementary file 1 — Supplemental Material [file 41409_2020_1129_MOESM1_ESM.docx]

**SUPPLEMENTARY INFORMATION**

**ELN risk stratification and outcomes in secondary and therapy-related AML patients following allogeneic stem cell transplantation,** Jentzsch *et al*.

**Patients also reported in publications by the EBMT**

Leipzig University Hospital is an active European Society of Blood and Marrow Transplantation (EBMT) center that registers and updates transplanted patients in the EBMT registry on a regular basis. Subsequently, patients included in this analysis have also been included in previous EBMT publications.

With regards to EBMT studies on s/tAML up to 273 of the here presented patients match the inclusion criteria by Schmaelter *et al*^1^ and were also reported in this study. Up to 203 patients of the here presented patients match the inclusion criteria in the manuscript by Sengsayadeth *et al*^2^ and were also reported in this study.

**Incidences of primary neoplasms**

Prior lymphomas were multiple myeloma (n=5), chronic lymphocytic leukemia (n=4), follicular lymphoma (n=2), marginal zone lymphoma (n=2), diffuse large B-cell lymphoma (n=1), Morbus Hodgkin, and hairy cell leukemia (n=1) while prior solid tumors were breast cancer (n=14), female genital tumors (n=6), gastrointestinal tumors (n=7), seminomas (n=5), upper respiratory tract tumors (n=2), bronchial carcinoma (n=1), bladder carcinoma (n=1), and histiocytoma (n=1).

**Further patients’ characteristics according to disease origin**

Additional clinical, genetic, molecular, and flow cytometry information at diagnosis for the whole patient cohort are shown in Supplementary Table S1. Hematopoietic stem cell transplantation (HSCT) related information for patients in the outcome set are shown in Supplementary Table S2.

**Induction therapy protocols of patients in the outcome set**

In the subgroup of acute myeloid leukemia (AML) patients younger than 60 years at diagnosis (n=283), 264 patients received chemotherapy according to the AML 2002 study (OSHO #061),^3^ four patients received chemotherapy within the Ratify trial,^4^ one patient received 7+3 with Midostaurin, two patients were treated within the Quantum first trial (ClinicalTrials.gov Identifier: NCT02668653), three patients received sequential Azacitidine and chemotherapy, seven patients were treated in the AMLCG 2008 study (ClinicalTrials.gov Identifier: NCT01382147), one patient received Azacitidine alone, and one patient was diagnosed with AML as a child and treated within the AML BFM-2014 study.^5^

Among AML patients older than 60 years at diagnosis (n=251), 214 patients were treated within the AML 2004 study (OSHO #069),^6^ 19 patients were treated within the OSHO #083 protocol, 13 patients received sequential Azacitidine and chemotherapy, two patients were treated within the Quantum first trial (ClinicalTrials.gov Identifier: NCT02668653), two patients were treated in the AMLCG 2008 study (ClinicalTrials.gov Identifier: NCT01382147) and one patient received Azacitidine alone.

**Allogeneic HSCT**

The majority of patients (n=379; 71%) received non-myeloablative (NMA) peripheral blood HSCT with 3x30 mg/m^2^ Fludarabine and 2 Gy (five patients received 3x30 mg/m^2^ Fludarabine and 3 Gy and five patients received 2 Gy alone) total body irradiation (TBI). 142 patients (27%) received myeloablative conditioning (MAC) consisting of 2x60 mg/kg body weight cyclophosphamide and 12 Gy total body irradiation and thirteen patients (2%) received reduced intensity conditioning (RIC) within the MC-FludT.14/L trial (EudraCT Number 2008-002356-18).

As patients after RIC and MAC-HSCT had comparable and significantly longer overall survival (OS) than patients after NMA-HSCT, they were analyzed together (Supplementary Figure S2).

**Prevention of graft-versus-host disease**

Prevention of graft-versus-host disease (GvHD) was different according to the conditioning regimes used. All patients receiving MAC or RIC were treated with cyclosporine A (CyA), starting intravenously with 5 mg/kg body weight (BW) in two daily doses from day -1. Blood levels of CyA were measured from day 0 and doses were adjusted for target levels of 200 ng/ml. Patients also received methotrexate 15 mg intravenously on days +1, +3, +6, and +11 after HSCT. Furthermore, patients with an unrelated donor additionally received *in vivo* T-cell depletion with thymoglobulin 2 mg/kg BW per day on days -3 to -1.

All patients with NMA-HSCT received a starting dose of 5 mg/kg BW CyA in two daily doses from day -1. Blood levels of CyA were measured from day 0 and doses were adjusted for target levels of 200 ng/ml. Additionally, patients with NMA conditioning received mycophenolate mofetil (MMF) 3 g per day in three daily doses if receiving unrelated HSCT or 2 g per day in two daily doses if receiving related HSCT. CyA was reduced starting on day +84 or day +180 following related or unrelated HSCT, respectively, and MMF was stopped at day +28 following related HSCT and tapered from days +40 to +96 following unrelated HSCT.^7^

For all patients after MAC, RIC or NMA-HSCT, immunosuppression was prolonged or extended with systemic steroids in cases of GvHD (grade > 2 according to Glucksberg grading system^8^) or rapidly reduced in patients who relapsed (≥ 5% blasts in bone marrow). Patients were evaluated for incidence of acute GvHD (aGvHD) and chronic GvHD (cGvHD), using established criteria of the Glucksberg grading system.^8^ Requirement for aGvHD was engraftment while requirement for cGvHD was engraftment and survival for at least 100 days after HSCT.

**Definition of complete remission**

CR was defined as the presence of <5% of blasts in bone marrow (BM), neutrophils >1.0 x 10^9^/L, platelets >100 x 10^9^/L, absence of blasts with Auer rods, independence of blood transfusion and no extramedullary disease.^9^ CR with incomplete peripheral recovery (CRi) was defined as CR with platelets <100 x 10^9^/L or neutrophils <1.0 x 10^9^/L. In patients receiving allogeneic HSCT, the presence of CR or CRi was confirmed within 28 days prior to HSCT by bone marrow and peripheral blood analysis.

**Statistical analyses and definition of clinical endpoints**

OS was calculated from HSCT until death from any cause. The competing risks cumulative incidence of relapse (CIR) and NRM were calculated from HSCT to relapse or death, respectively, using the Fine and Gray method.^10^ Associations with baseline clinical, demographic, and molecular features were compared using the Kruskal-Wallis-Test and Fisher’s exact tests for continuous and categorical variables, respectively. Survival estimates were calculated using the Kaplan-Meier method and groups were compared using the log-rank test.

**Multivariate analyses**

We constructed a two multivariable proportional hazard model for cumulative incidence of relapse (CIR), non-relapse mortality (NRM) and OS to evaluate the impact of disease origin in patients treated with allogeneic HSCT by backward adjusting for other variables. The following variables were considered for multivariable analyses: sex, disease origin (*de novo* vs secondary), European LeukemiaNet (ELN) risk, mutation status of the tyrosine kinase domain of the *FLT3* gene (*FLT3*-TKD), age at HSCT, disease status at HSCT (CR *vs* CRi), pre-HSCT MRD status (positive *vs* negative), cytomegalovirus (CMV) status of recipient and donor (high risk [+/-] *vs* all others), donor type (matched related *vs* matched unrelated *vs* mismatched unrelated) and sex of the donor. Of these, variables significant at α=.10 in univariable analyses were considered for multivariable analyses. For all endpoints, hazard/odds ratios with their corresponding 95% confidence intervals are indicated for every significant prognostic factor of the final model.

**Immunophenotype**

s/tAML patients presented with a distinct immunophenotype with a higher CD34+/CD38- cell burden (*P*<.001) but lower expression of the immature antigen CD117 (*P*=.006), lower expression of myeloid antigens (i.e. CD13, *P*<.001; CD33, *P*<.001; CD15, *P*<.001; CD64, *P*<.001 and CD65, *P*=.008), lower expression of pan-leukocyte antigens (i.e. CD45, *P*<.001 and CD38, *P*<.001) and higher expression of T-cell antigens (i.e. CD2, *P*=.002; CD7, *P*=.002 and CD56, *P*<.001, Supplementary Table S1).

**Multivariate Analyses for patients receiving NMA or RIC- or MAC-HSCT**

Also in separate multivariate analyses for patients receiving NMA-HSCT and patients receiving RIC- or MAC-HSCT, disease origin did not impact CIR or OS (Supplementary Table S4). Similar to the whole patient cohort, only the ELN risk classification at diagnosis and the pre-HSCT MRD status were significant prognostic factors for both end-points. After RIC or MAC-HSCT, s/tAML patients had a higher NRM after adjustment for age at HSCT while only a mismatched donor and lacking peripheral blood count recovery associated with higher NRM after NMA-HSCT.

**Pre-HSCT MRD status**

The amount of pre-HSCT MRD-positive or MRD-negative patients did not differ significantly between *de novo* and s/tAML individuals in the whole patient cohort (Supplementary Table S3) as well as in separate analyses for the three ELN2017 risk groups (Supplementary Table S5). While we observed a significant impact on CIR after HSCT for the pre-HSCT MRD status in both *de novo* and s/tAML patients (*P*<.001 and *P*=.004, respectively, Supplementary Figure S5), MRD-positive patients had dismal outcomes, irrespective of disease origin (Supplementary Figure S4). Interestingly, in MRD-negative AML patients the time to relapse was significantly shorter in s/tAML compared to *de novo* AML patients (*P*=.03), but not distinct in MRD-positive individuals (*P*=.47).

**SUPPLEMENTARY REFERENCES**

1 Schmaelter AK, Labopin M, Socié G, Itälä-Remes M, Blaise D, Yakoub-Agha I *et al.* Inferior outcome of allogeneic stem cell transplantation for secondary acute myeloid leukemia in first complete remission as compared to de novo acute myeloid leukemia. *Blood Cancer J* 2020; **10**: 10–26.

2 Sengsayadeth S, Gatwood KS, Boumendil A, Labopin M, Finke J, Ganser A *et al.* Conditioning intensity in secondary AML with prior myelodysplastic syndrome/myeloproliferative disorders: An EBMT ALWP study. *Blood Adv* 2018; **2**: 2127–2135.

3 Büchner T, Schlenk RF, Schaich M, Doḧner K, Krahl R, Krauter J *et al.* Acute Myeloid Leukemia (AML): Different treatment strategies versus a common standard arm - Combined prospective analysis by the German AML Intergroup. *J Clin Oncol* 2012; **30**: 3604–3610.

4 Stone RM, Mandrekar SJ, Sanford BL, Laumann K, Geyer S, Bloomfield CD *et al.* Midostaurin plus chemotherapy for acute myeloid leukemia with a FLT3 Mutation. *N Engl J Med* 2017; **377**: 454–464.

5 Creutzig U, Zimmermann M, Bourquin JP, Dworzak MN, Fleischhack G, Graf N *et al.* Randomized trial comparing liposomal daunorubicin with idarubicin as induction for pediatric acute myeloid leukemia: Results from study AML-BFM 2004. *Blood* 2013; **122**: 37–43.

6 Niederwieser D, Hoffmann VS, Pfirrmann M, Al-Ali HK, Schwind S, Vucinic V *et al.* Comparison of Treatment Strategies in Patients over 60 Years with AML: Final Analysis of a Prospective Randomized German AML Intergroup Study. [abstract]. In: *Blood*. 2016, p 1066.

7 Niederwieser D, Maris M, Shizuru JA, Petersdorf E, Hegenbart U, Sandmaier BM *et al.* Low-dose total body irradiation (TBI) and fludarabine followed by hematopoietic cell transplantation (HCT) from HLA-matched or mismatched unrelated donors and postgrafting immunosuppression with cyclosporine and mycophenolate mofetil (MMF) can induce dura. *Blood* 2003; **101**: 1620–1629.

8 Glucksberg H, Storb R, Fefer A, Buckner CD, Neiman PE, Clift RA *et al.* Clinical manifestations of graft-versus-host disease in human recipients of marrow from HL-A-matched sibling donors. 1974; : 295–304.

9 Döhner H, Estey EH, Amadori S, Appelbaum FR, Büchner T, Burnett AK *et al.* Diagnosis and management of acute myeloid leukemia in adults: Recommendations from an international expert panel, on behalf of the European LeukemiaNet. *Blood* 2010; **115**: 453–474.

10 Gray RJ. A Class of K-Sample Tests for Comparing the Cumulative Incidence of a Competing Risk. *Ann Stat* 1988; **16**: 1141–1154.

**Supplemental Tables**

**Table S1: Clinical and genetic characteristics for all patients according to disease origin (*de novo* vs secondary and *de novo* *vs* treatment related), n=644**

|  | ***de novo***  **AML**  **n=416** | **secondary**  **AML**  **n=171** | ***P*** | **treatment related**  **AML**  **n=57** | ***P*** |
| --- | --- | --- | --- | --- | --- |
| Age at diagnosis, years  median  range | 56.0  14.3-76.5 | 62.3  27.1-74.9 | <.001 | 61.6  31.7-74.9 | .006 |
| Sex, n (%)  male  female | 204 (49)  212 (51) | 107 (63)  64 (37) | .003 | 23 (40)  34 (60) | .26 |
| Hemoglobin, g/dL  median  range | 9  3.2-15.7 | 8.9  5.4-15 | .27 | 8.4  5.5-14.2 | .19 |
| Platelet count, x 10^9^/L  median  range | 65  2-950 | 64  1-547 | .61 | 43  3-287 | .07 |
| WBC, x 10^9^/L  median  range | 8.6  0.5-385 | 5.7  0.1-432 | .03 | 3.7  0.8-130 | .05 |
| Blood blasts, %  median  range | 24  0-98 | 10  0-97 | <.001 | 15  0-89 | .22 |
| BM blasts, %  median  range | 60  0-100 | 32  0-93 | <.001 | 55  3-95 | .53 |
| BM CD34+/CD38- burden, %  median  range | 0.5  0-75 | 1.5  0-89 | <.001 | 0.8  0-64 | .05 |
| Normal karyotype, n (%)  absent  present | 192 (52)  180 (48) | 97 (61)  61 (39) | .05 | 39 (68)  18 (32) | .02 |
| ELN2017 group, n (%)  favorable  intermediate  adverse | 97 (33)  84 (28)  115 (39) | 12 (12)  35 (34)  56 (54) | <.001 | 5 (13)  10 (26)  24 (62) | .01 |
| *NPM1*, n (%)  wild-type  mutated | 217 (71)  90 (29) | 95 (88)  13 (12) | <.001 | 32 (80)  8 (29) | .27 |
| *CEBPA*, n (%)  wild-type  mutated | 232 (88)  31 (12) | 70 (86)  11 (14) | .70 | 24 (89)  3 (11) | 1 |
| *FLT3*-ITD, n (%)  absent  present | 224 (72)  88 (28) | 99 (91)  10 (9) | <.001 | 14 (87)  5 (13) | .05 |
| *FLT3*-TKD, n (%)  wild-type  mutated | 248 (87)  37 (13) | 100 (98)  2 (2) | .001 | 30 (91) 3 (9) | .78 |
| *RUNX1*, n (%)  wild-type  mutated | 61 (85)  11 (15) | 28 (85)  5 (15) | 1 | 6 (86)  1 (14) | 1 |
| *ASXL1*, n (%)  wild-type  mutated | 63 (88)  9 (13) | 26 (79)  7 (21) | .26 | 6 (86)  1 (14) | 1 |
| *TP53*, n (%)  wild-type  mutated | 64 (89)  8 (11) | 29 (88)  4 (12) | 1 | 6 (86)  1 (14) | .59 |
| *Abbreviations: ASXL1, additional sex combs-like 1 gene; BM, bone marrow; BAALC, brain and acute leukemia cytogenetic gene; CEBPA, CCAAT/enhancer-binding protein alpha gene; ELN, European Leukemia Net; FLT3-ITD, internal tandem duplication of the FLT3 gene; Hb, hemoglobin; MN1, meningioma 1 gene; NPM1, nucleophosmin 1 gene; PB, peripheral blood; RUNX1, Runt-related transcription factor 1 gene; TP53, tumor protein 53 gene; WBC, white blood count.* | | | | | |

**Table S2:** **Additional clinic, genetic and flow cytometry characteristics at diagnosis for all patients (“association set”) according disease origin (*de novo* *vs* secondary and *de novo vs* treatment related AML), n=644.**

|  | **all patients**  **n=644** | ***de novo***  **AML**  **n=416** | **secondary or treatment related AML**  **n=228** | ***P*** | **secondary**  **AML**  **n=171** | ***P***  **(de *novo* vs sAML)** | **treatment related**  **AML**  **n=57** | ***P***  **(de *novo* vs tAML)** |
| --- | --- | --- | --- | --- | --- | --- | --- | --- |
| Additional clinical Information at diagnosis | | | | | | | | |
| FAB type, n (%)  M0  M1  M2  M4  M4eo  M5  M6  M7 | 17  63  240  80  20  50  14  10 | 14 (4)  54 (14)  172 (46)  62 (16)  20 (5)  40 (11)  12 (3)  6 (2) | 3 (3)  9 (8)  68 (60)  18 (16)  0 (0)  10 (9)  2 (2)  4 (4) | .77  .08  .01  1  .006  .72  .54  .25 | 2 (3)  6 (8)  52 (67)  9 (12)  0 (0)  5 (6)  1 (1)  3 (4) | 1  .14  .001  .39  .03  .30  .71  .19 | 1 (3)  3 (9)  16 (46)  8 (23)  0 (0)  5 (14)  1 (3)  1 (3) | 1  .45  1  .35  .40  .57  1  .46 |
| Additional genetic information at diagnosis | | | | | | | | |
| CBF-AML, n (%)  absent  present | 538  30 | 333 (92)  30 (8) | 205 (100)  0 (0) | <.001 | 150 (100)  0 (0) | <.001 | 54 (100)  0 (0) | .01 |
| Trisomy 8, n (%)  absent  present | 511  68 | 333 (90)  38 (10) | 178 (86)  30 (14) | .14 | 128 (84)  25 (16) | .06 | 49 (91)  5 (9) | 1 |
| del(5)/del(5q), n (%)  absent  present | 518  64 | 343 (92)  29 (8) | 175 (83)  35 (17) | .001 | 131 (85)  24 (16) | .01 | 43 (80)  11 (20) | .01 |
| del(7)/del(7q), n (%)  absent  present | 499  88 | 332 (89)  42 (11) | 167 (78)  46 (22) | .001 | 129 (82)  28 (18) | .05 | 37 (67)  18 (33) | <.001 |
| Monosomal karyotype, n (%)  absent  present | 490  78 | 321 ((89)  41 (11) | 169 (82)  37 (18) | .03 | 132 (87)  19 (13) | .76 | 37 (69)  17 (31) | <.001 |
| Complex karyotype, n (%)  absent  present | 476  86 | 310 (87)  48 (13) | 166 (81)  38 (19) | .11 | 129 (85)  23 (15) | .68 | 36 (71)  15 (29) | .006 |
| *SRSF2*, n (%)  wild-type  mutated | 134  19 | 91 (91)  9 (9) | 43 (81)  10 (19) | .66 | 33 (77)  10 (23) | .03 | 10 (100)  0 (0) | 1 |
| *U2AF1*-TKD, n (%)  wild-type  mutated | 108  4 | 69 (96)  3 (4) | 39 (98)  1 (3) | 1 | 32 (97)  1 (3) | 1 | 7 (100)  0 (0) | 1 |
| *SF3B1*, n (%)  wild-type  mutated | 102  10 | 65 (90)  7 (10) | 37 (93)  3 (8) | 1 | 31 (94)  2 (6) | .72 | 6 (86)  1 (14) | .54 |
| *ZRSR2*, n (%)  wild-type  mutated | 108  4 | 69 (96)  3 (4) | 39 (98)  1 (3) | 1 | 32 (97)  1 (3) | 1 | 7 (100)  0 (0) | 1 |
| *BCOR*, n (%)  wild-type  mutated | 96  16 | 62 (86)  10 (14) | 34 (85)  6 (15) | 1 | 28 (85)  5 (15) | 1 | 6 (86)  1 (14) | 1 |
| *STAG2*, n (%)  wild-type  mutated | 105  7 | 68 (94)  4 (6) | 37 (93)  3 (8) | .70 | 31 (94)  2 (6) | 1 | 6 (86)  1 (14) | .38 |
| *EZH2*, n (%)  wild-type  mutated | 106  6 | 67 (93)  5 (7) | 39 (98)  1 (3) | .42 | 32 (97)  1 (3) | .66 | 7 (100)  0 (0) | 1 |
| *RAS* pathway mutations, n (%)  absent  present | 88  24 | 53 (74)  19 (26) | 35 (88)  5 (13) | .10 | 29 (88)  4 (12) | .13 | 6 (86)  1 (14) | .67 |
| *SETBP1*, n (%)  wild-type  mutated | 110  2 | 71 (99)  1 (1) | 39 (98)  1 (3) | 1 | 32 (97)  1 (3) | .53 | 7 (100)  0 (0) | 1 |
| *IDH1*, n (%)  wild-type  mutated | 190  20 | 123 (90)  13 (10) | 67 (91)  7 (9) | 1 | 52 (93)  4 (7) | .78 | 15 (83)  3 (17) | .40 |
| *IDH2*, n (%)  wild-type  mutated | 181  31 | 117 (86)  19 (14) | 64 (84)  12 (16) | .84 | 49 (86)  8 (14) | 1 | 15 (79)  4 (21) | .49 |
| *DNMT3A*, n (%)  wild-type  mutated | 136  24 | 93 (82)  20 (18) | 43 (91)  4 (9) | .15 | 34 (929  3 (8) | .20 | 9 (90)  1 (10) | 1 |
| *TET2*, n (%)  wild-type  mutated | 90  22 | 58 (81)  14 (19) | 32 (80)  8 (20) | 1 | 28 (85)  5 (15) | .79 | 4 (57)  3 (43) | .17 |
| *JAK2*, n (%)  wild-type  mutated | 103  9 | 72 (100)  0 (0) | 31 (78)  9 (23) | <.001 | 24 (73)  9 (27) | <.001 | 7 (100)  0 (0) | 1 |
| *CUX1*, n (%)  wild-type  mutated | 106  6 | 66 (92)  6 (8) | 40 (100)  0 (0) | .09 | 33 (100)  0 (0) | .17 | 7 (100)  0 (0) | 1 |
| **Immunophenotype** | | | | | | | | |
| BM CD117 expression, %  median  range | 33  0-96 | 37  0.5-95 | 26  0-96 | .006 | 25  0-96 | .003 | 32  0.5-89 | .50 |
| BM CD38 expression, %  median  range | 71  0.5-98 | 79  0.5-98 | 58  4-98 | <.001 | 56  4-98 | <.001 | 69  21-97 | .08 |
| BM CD45 expression, %  median  range | 92  6-100 | 94  6-100 | 86  23-100 | <.001 | 84  23-100 | <.001 | 92  39-99 | .28 |
| BM CD11b expression, %  median  range | 16  0.5-97 | 16  0.5-97 | 15  1-93 | .73 | 16  1-71 | .88 | 13  1-93 | .60 |
| BM CD13 expression, %  median  range | 56  0.5-97 | 61  0.5-97 | 42  3-96 | <.001 | 33  3-96 | <.001 | 58  9-95 | .64 |
| BM CD15 expression, %  median  range | 27  2-97 | 34  2-97 | 19  2-94 | .<.001 | 19  2-91 | <.001 | 25  3-94 | .16 |
| BM CD33 expression, %  median  range | 63  1-96 | 72  1-98 | 47  3-96 | <.001 | 41  3-95 | <.001 | 54  9-96 | .14 |
| BM CD65 expression, %  median  range | 17  0.5-93 | 20  0.5-93 | 11  0.5-91 | .008 | 10  0.5-82 | .007 | 13  0.5-19 | .32 |
| BM CD14 expression, %  median  range | 2  0.5-74 | 2  0.5-70 | 2  0.5-74 | .82 | 2  0.5-59 | .91 | 3  0.5-74 | .45 |
| BM CD64 expression, %  median  range | 17  0-98 | 20  0-98 | 8  0.5-92 | <.001 | 6  0.5-86 | <.001 | 11  0.5-92 | .11 |
| BM CD61 expression, %  median  range | 5  0.5-72 | 3  0.5-72 | 7  0.5-56 | <.001 | 9  0.5-56 | <.001 | 5  0.5-24 | .32 |
| BM Glykophorin A expression, %  median  range | 11  0-90 | 8  0-90 | 15  0-73 | <.001 | 15  0-72 | <.001 | 12  2-73 | .04 |
| BM CD2 expression, %  median  range | 15  1-97 | 13  1-97 | 20  2-93 | <.001 | 22  2-93 | <.001 | 13  3-62 | .65 |
| BM CD7 expression, %  median  range | 17  1-94 | 14  1-94 | 23  2-93 | .002 | 23  2-76 | .001 | 17  3-93 | .39 |
| BM CD56 expression, %  median  range | 10  0-97 | 7  0-97 | 13  0.5-93 | .002 | 13  0.5-93 | .001 | 16  1-84 | .02 |
| *Abbreviations: BCOR, BCL-6-Corepressor gene; BM, bone marrow; CBF, core binding factor; CUX, cut-like homeobox 1 gene; del, deletion; DNMT3A, DNA-methyltransferase 3A gene; EZH2, Enhancer of zeste homolog 2 gene; FAB, french american british; FLT3-TKD, tyrosine kinase domain of the FLT3 gene; IDH1, isocitrat dehydrogenase 1 gene; IDH2, isocitrat dehydrogenase 2 gene; JAK2, janus kinase 2 gene; RAS, rat sarcoma gene; SETBP1, SET Binding Protein 1 gene; SF3B1, Splicing Factor 3b Subunit 1; SRSF2, Serine And Arginine Rich Splicing Factor 2 gene; STAG2, Stromal Antigen 2 gene; TET2, Ten-Eleven Translocation-2 gene; U2AF1, U2 Small Nuclear RNA Auxiliary Factor 1 gene; ZRSR2, Zinc finger (CCCH type), RNA-binding motif and serine/arginine rich 2 gene.* | | | | | | | | |

**Table S3:** **HSCT-associated characteristics for patients in the outcome according to disease origin (*de novo* *vs* secondary and *de novo vs* treatment related AML), n=534**

|  | **all patients**  **n=534** | ***de novo***  **AML**  **n=356** | **secondary or treatment related AML**  **n=178** | ***P*** | **secondary**  **AML**  **n=133** | ***P***  **(*de novo vs* sAML)** | **treatment related**  **AML**  **n=45** | ***P***  **(*de novo vs* tAML)** |
| --- | --- | --- | --- | --- | --- | --- | --- | --- |
| Conditioning regimen, n (%)  MAC  RIC  NMA | 142  13  379 | 118 (33)  9 (3)  229 (64) | 24 (13)  4 (2)  150 (84) | .03 | 15 (11)  4 (3)  114 (86) | <.001 | 9 (20)  0 (0)  36 (80) | .11 |
| Number of remission at HSCT, n (%)  CR/CRi1  CR/CRi2  CR/CRi3 | 426  114  4 | 266 (75)  87 (24)  3 (1) | 150 (84)  27 (15)  1 (1) | .03 | 113 (85)  19 (14)  1 (1) | .03 | 37 (82)  8 (18)  0 (0) | .55 |
| Remission status at HSCT, n (%)  CR  CRi | 451  83 | 312 (88)  44 (12) | 139 (78)  39 (22) | .005 | 100 (75)  33 (25) | <.001 | 39 (87)  6 (13) | .81 |
| HCT-CI Score, n (%)  0  1/2  ≥ 3 | 237  140  152 | 171 (48)  98 (28)  85 (24) | 66 (38)  42 (24)  67 (38) | .003 | 63 (48)  36 (27)  32 (24) | 1 | 3 (7)  6 (14)  35 (80) | <.001 |
| donor type, n (%)  HLA matched related  HLA matched unrelated  HLA mismatched unrelated | 121  306  107 | 94 (26)  197 (56)  65 (18) | 27 (15)  109 (61)  42 (24) | .009 | 20 (15)  82 (62)  31 (23) | .02 | 7 (16)  27 (60)  11 (24) | .23 |
| donor & recipient sex, n (%)  no female into male  female into male | 449  77 | 296 (84)  55 (16) | 153 (87)  22 (13) | .36 | 113 (86)  19 (14) | .78 | 40 (93)  3 (7) | .17 |
| CMV status, n (%)  recipient + / donor –  all others | 197  338 | 127 (36)  222 (64) | 70 (40)  106 (60) | .45 | 51 (37)  81 (63) | .65 | 19 (43)  25 (57) | .41 |
| aGvHD ≥ grade 2, n (%)  absent  present | 334  130 | 230 (75)  77 (25) | 104 (66)  53 (34) | .06 | 78 (67)  39 (33) | .09 | 26 (65)  14 (35) | .18 |
| cGvHD, n (%)  absent  limited  extended | 143  50  174 | 92 (37)  38 (15)  116 (47) | 51 (42)  12 (10)  58 (49) | .31 | 37 (43)  6 (7)  43 (50) | .12 | 14 (40)  6 (17)  15 (43) | .86 |
| **Pre-HSCT MRD** | | | | | | | | |
| Pre-HSCT MRD, n (%)  negative  positive | 144  100 | 98 (60)  66 (40) | 46 (58)  34 (43) | .78 | 30 (54)  26 (46) | .44 | 16 (67)  8 (33) | .66 |
| *Abbreviations: aGvHD, acute graft versus host disease; cGvHD, chronic graft versus host disease; CMV, cytomegalovirus; CR, complete remission; CRi, CR with incomplete peripheral recovery; HLA, human leukocyte antigen; HCT-CI, hematpopietic cell transplantation comorbidity index; HSCT, hematopoietic stem cell transplantation; MRD, measurable residual disease.* | | | | | | | | |

**Supplementary Table S4: Multivariate Analyses for patients receiving NMA-HSCT and patients receiving RIC- or MAC-HSCT.**

|  | Cumulative Incidence of Relapse | | Cumulative Incidence of Non-relapse Mortality | | Overall Survival | |
| --- | --- | --- | --- | --- | --- | --- |
|  | **HR* (95% CI)** | ***P*** | **HR* (95% CI)** | ***P*** | **OR** (95% CI)** | ***P*** |
| Models for patients receiving NMA-HSCT | | | | | | |
| ELN2017 risk  (adverse *vs* intermediate *vs* favorable) | 1.76 (1.22 - 2.54) | .003 | - | - | 0.70 (0.52 - 0.94) | .02 |
| Donor type  (mismatched *vs* matched unrelated *vs* related) | - | - | 1.48 (1.07 - 2.05) | .02 | - | - |
| Remission status at HSCT  (CR *vs* CRi) | - | - | 0.46 (0.29 - 0.75) | .002 | - | - |
| Pre-HSCT MRD status  (positive *vs* negative) | 3.04 (1.72 - 5.37) | <.001 |  |  | 0.55 (0.34 – 0.89) | .02 |
| Models for patients receiving RIC or MAC-HSCT | | | | | | |
| ELN2017 risk  (adverse *vs* intermediate *vs* favorable) | - | - | - | - | 0.46 (0.28 - 0.74) | .02 |
| Disease origin  (*de novo* *vs* secondary) | - | - | 0.30 (0.12 – 0.76) | .01 | - | - |
| Pre-HSCT MRD status  (positive *vs* negative) | 6.98 (1.79 – 27.2) | .005 | - | - | - | - |
| Age at HSCT | - | - | 1.07 (1.03 - 1.12) | .002 | 0.95 (0.91 – 0.99) | .008 |
| Abbreviations: AML, acute myeloid leukemia; CI, confidence interval; CR, complete remission; CRi, complete remission with incomplete peripheral recovery; ELN, European LeukemiaNet; HSCT, hematopoietic stem cell transplantation; MRD, measurable residual disease.  *HR, hazard ratio, <1 (>1) indicate lower (higher) risk of relapse for the first category listed for the dichotomous variables.  **OR, odds ratio, <1 (>1) indicate lower (higher) chance of survival for the first category listed for the dichotomous variables.  Variables considered in the models were those significant at α=0.10 in univariable analyses.  For cumulative incidence of relapse endpoint, variables considered were: in patients receiving NMA-HSCT: ELN risk group and pre-HSCT MRD status; in patients receiving RIC/MAC HSCT: ELN2017 risk group and pre-HSCT MRD status.  For non-relapse mortality endpoint, variables considered were: in patients receiving NMA-HSCT: donor type (mismatched unrelated *vs* matched unrelated *vs* related) and remission status at HSCT (CR *vs* CRi); in patients receiving RIC/MAC HSCT: disease origin (*de novo vs* secondary) and age at HSCT.  For OS endpoint, variables considered were: in patients receiving NMA-HSCT: sex, ELN2017 risk group, donor sex (female into male *vs* all others), remission status at HSCT (CR *vs* CRi) and pre-HSCT MRD status; in patients receiving RIC/MAC HSCT: ELN risk group, disease origin (*de novo vs* s/tAML), and age at HSCT. | | | | | | |

**Table S5: Clinical and genetic characteristics within the three ELN risk groups according to disease origin (*de novo* *vs* secondary or treatment related).**

|  | **all patients** | ***de novo***  **AML** | **secondary or treatment related AML** | ***P*** |
| --- | --- | --- | --- | --- |
| **ELN favorable risk** | | | | |
| Age at diagnosis, years  median  range | 55.8  14.3-73.9 | 54.9  14.3-73.9 | 64.1  45.4-71.3 | .02 |
| Platelet count, x 10^9^/L  median  range | 76  3-276 | 71  6-276 | 92  3-179 | .63 |
| WBC, x 10^9^/L  median  range | 17.1  1-162 | 17.2  1-162 | 16  1-90 | .56 |
| BM blasts, %  median  range | 52  14-100 | 55  22-100 | 42  14-88 | .08 |
| BM CD34+/CD38- burden, %  median  range | 0.1  0-21 | 0.1  0-21 | 0.1  0-10 | .99 |
| Normal karyotype, n (%)  absent  present | 42  66 | 40 (44)  51 (56) | 2 (12)  15 (88) | .01 |
| CBF AML, n (%)  absent  present | 78  30 | 61 (67)  30 (33) | 17 (100)  0 (0) | .003 |
| *NPM1*, n (%)  wild-type  mutated | 30  78 | 29 (32)  62 (68) | 1 (6)  16 (94) | .04 |
| *FLT3*-ITD, n (%)  absent  present | 89  20 | 72 (78)  20 (22) | 17 (100)  0 (0) | .04 |
| *FLT3*-TKD, n (%)  wild-type  mutated | 83  18 | 68 (80)  17 (20) | 15 (94)  1 (6) | .29 |
| Pre-HSCT MRD, n (%)  negative  positive | 41  28 | 35 (63)  21 (38) | 6 (46)  7 (54) | .35 |
| **ELN intermediate risk** | | | | |
| Age at diagnosis, years  median  range | 59.6  18.5-76.5 | 56.9  18.5-76.5 | 61.3  27.1-74.7 | .02 |
| Platelet count, x 10^9^/L  median  range | 68  1-517 | 64  7-501 | 84  1-517 | .24 |
| WBC, x 10^9^/L  median  range | 4.1  0.7-295 | 4.3  0.7-198 | 3.7  0.8-295 | .91 |
| BM blasts, %  median  range | 52  0-95 | 61  0-95 | 31  11-93 | .003 |
| BM CD34+/CD38- burden, %  median  range | 0.5  0-89 | 0.5  0-60 | 1  0-89 | .18 |
| Normal karyotype, n (%)  absent  present | 48  75 | 26 (33)  52 (67) | 22 (49)  23 (51) | .12 |
| *NPM1*, n (%)  wild-type  mutated | 115  14 | 71 (85)  13 (15) | 44 (98)  1 (2) | .03 |
| *FLT3*-ITD, n (%)  absent  present | 98  31 | 58 (69)  26 (31) | 40 (89)  5 (11) | .02 |
| *FLT3*-TKD, n (%)  wild-type  mutated | 112  10 | 73 (90)  8 (10) | 39 (95)  2 (5) | .49 |
| *DNMT3A*, n (%)  absent  present | 21  9 | 9 (53)  8 (47) | 12 (92)  1 (8) | .04 |
| *JAK2*, n (%)  wild-type  mutated | 23  7 | 17 (100)  0 (0) | 6 (46)  7 (54) | .001 |
| Pre-HSCT MRD, n (%)  negative  positive | 35  26 | 24 (60)  16 (40) | 11 (52)  10 (48) | .60 |
| **ELN adverse risk** | | | | |
| Age at diagnosis, years  redian  Range | 59.3  23.3-75.8 | 55.2  23.3-75.8 | 62.1  31.9-74.9 | .008 |
| Plamelet count, x 10^9^/L  median  range | 52  2-950 | 67  2-950 | 38  3-488 | .03 |
| WBC, x 10^9^/L  median  range | 4.3  0.1-385 | 4.5  0.6-385 | 3.6  0.1-81.9 | .16 |
| BM blasts, %  median  range | 50  0-95 | 50  13.6-95 | 32.5  0-95 | .006 |
| BM CD34+/CD38- burden, %  median  range | 2  0-75 | 1.5  0-75 | 2.9  0-64 | .05 |
| Normal karyotype, n (%)  absent  present | 176  18 | 99 (88)  14 (12) | 77 (95)  4 (5) | .09 |
| Complex karyotype, n (%)  absent  present | 95  83 | 60 (57)  45 (43) | 35 (48)  38 (52) | .29 |
| Monosomal karyotype, n (%)  absent  present | 105  75 | 67 (63)  39 (37) | 38 (51)  36 (49) | .13 |
| *NPM1*, n (%)  wild-type  mutated | 143  4 | 85 (96)  4 (4) | 58 (100)  0 (0) | .15 |
| *FLT3*-ITD, n (%)  absent  present | 128  20 | 73 (81)  17 (19) | 55 (95)  3 (5) | .03 |
| *FLT3*-TKD, n (%)  wild-type  mutated | 128  10 | 75 (89)  9 (11) | 53 (98)  1 (2) | .09 |
| *RUNX1*, n (%)  wild-type  mutated | 37  15 | 23 (70)  10 (30) | 14 (74)  5 (26) | 1 |
| *ASXL1*, n (%)  wild-type  mutated | 39  13 | 28 (85)  5 (15) | 11 (58)  8 (42) | .05 |
| *TP53*, n (%)  wild-type  mutated | 40  12 | 26 (79)  7 (21) | 14 (74)  5 (26) | .74 |
| Pre-HSCT MRD, n (%)  negative  positive | 36  34 | 25 (60)  17 (40) | 11 (39)  17 (61) | .14 |
| *Abbreviations: ASXL1, additional sex combs-like 1 gene; BM, bone marrow; CBF, core binding factor;; ELN, European LeukemiaNet; FLT3-ITD, internal tandem duplication of the FLT3 gene; MRD, measurable residual disease; NPM1, nucleophosmin 1 gene; PB, peripheral blood; RUNX1, Runt-related transcription factor 1 gene; TP53, tumor protein 53 gene; WBC, white blood count.* | | | | |

**Supplementary Figures**

**Supplementary Figure S1**

**Supplementary Figure S1: Outcome according to the used conditioning regimen (NMA *vs* RIC or MAC conditioning) of patients in the outcome set, n=534. (A)** Cumulative Incidence of Relapse, **(B)** Non-relapse Mortality and **(C)** Overall Survival.

**Supplementary Figure S2**

**Supplementary Figure S2: Overall Survival for *de novo* and s/tAML patients according to the applied conditioning regimen (MAC *vs* RIC *vs* NMA-HSCT)**

**Supplementary Figure S3**

**Supplementary Figure S3: Outcome according to disease origin (*de novo vs* secondary or treatment related AML) for patients transplanted in first CR/CRi in the outcome set. (A)** Cumulative Incidence of Relapse, **(B)** Non-relapse Mortality and **(C)** Overall Survival in all patients (n=416). **(D)** Cumulative Incidence of Relapse, **(E)** Non-relapse Mortality and **(F)** Overall Survival in all patients. for patients receiving NMA-HSCT (n=289) and **(G)** Cumulative Incidence of Relapse, **(H)** Non-relapse Mortality and **(I)** Overall Survival for patients receiving RIC- or MAC-HSCT (n=127).

**Supplementary Figure S4**

**Supplementary Figure S4: Outcome according disease origin in pre-HSCT MRD positive AML patients. (A)** Cumulative Incidence of Relapse and **(B)** Overall Survival.

**Supplementary Figure S5**

**
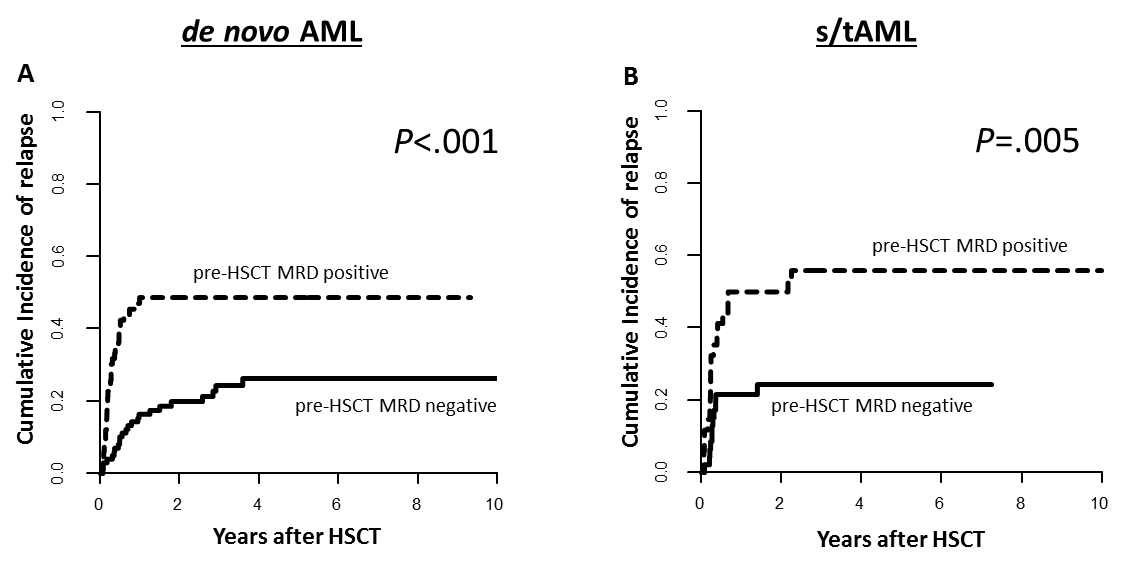
**

**Supplementary Figure S5: Cumulative Incidence of Relapse according to pre-HSCT MRD status in (A)** *de novo* and **(B)** secondary or treatment related AML patients.

**Supplementary Figure S6**

**Supplementary Figure S6: Cumulative Incidence of Relapse according to pre-HSCT MRD status in (A)** *de novo* or s/tAML patients receiving NMA-HSCT and **(B)** *de novo* or s/AML patients receiving RIC- or MAC-HSCT.

**Supplementary Figure S7**

**Supplementary Figure S7: CD33 expression levels at diagnosis in *de novo* AML, sAML and tAML patients.**
